# Microlens Fabrication by Replica Molding of Electro-Hydrodynamic Printing Liquid Mold

**DOI:** 10.3390/mi11020161

**Published:** 2020-02-03

**Authors:** Feiyu Fang, Xulei Tao, Xun Chen, Han Wang, Peixuan Wu, Jiarong Zhang, Jun Zeng, Ziming Zhu, Zhen Liu

**Affiliations:** 1 State Key Laboratory of Precision Electronic Manufacturing Technology and Equipment, School of Electromechanical Engineering, Guangdong University of Technology, Guangzhou 510006, China; 2 Guangdong Provincial Key Laboratory of Micro-Nano Manufacturing Technology and Equipment, School of Electromechanical Engineering, Guangdong University of Technology, Guangzhou 510006, China; 3 Guangdong Provincial Jihua Laboratory, Foshan 528000, China; 4 Department of Physics and Engineering, Frostburg State University, Frostburg, MD 21532, USA

**Keywords:** microlenses, replica molding, electrohydrodynamic printing, soft lithography

## Abstract

In this paper, we synergistically combine electrohydrodynamic (EHD) printing and replica molding for the fabrication of microlenses. Glycerol solution microdroplets was sprayed onto the ITO glass to form liquid mold by an EHD printing process. The liquid mold is used as a master to fabricate a polydimethylsiloxane (PDMS) mold. Finally, the desired micro-optical device can be fabricated on any substrate using a PDMS soft lithography mold. We demonstrate our strategy by generating microlenses of photocurable polymers and by characterizing their optical properties. It is a new method to rapidly and cost-effectively fabricate molds with small diameters by exploiting the advantages of EHD printing, while maintaining the parallel nature of soft-lithography.

## 1. Introduction

Microlens and microlens array (MLA) with diameters of a few to hundreds micrometers have wide applications in various fields, including artificial compound eyes [1], light-emitting devices [2], microfluidic system [3], sensors [4], and so on. These applications require the fabrication of microlens arrays on different substrates or even on curved surfaces, thus requiring achievable microlens fabrication processes. Various methods for the fabrication of microlens array have been developed [5], including, but not limited to photoresist reflow method [6], hot embossing [7], and femtosecond laser direct-write [8]. However, conventional manufacturing processes are difficult to achieve large-scale microlens array fabrication on different substrates, or limited by expensive equipment and complicated processes.

Inkjet printing and laser-induced transfer provide methods of fabricating microlens molds or directly fabricating microlens arrays [9,10]. The surface tension causes the resulting liquid droplets to be almost perfectly spherical, so that high optical quality microlens can be printed or replicated. However, the scale is still limited, which cannot meet the needs of some special applications. Moreover, inkjet printing is limited by the viscosity of the ink, making it difficult to print high viscosity materials [11].

The soft lithography method is a method of performing microstructure replication or patterning polymer droplets using a PDMS elastomer mold [12,13,14]. By patterning a photocurable prepolymer, a subsequent photocuring step can convert the droplet into a solid microlens, which is a method that enables rapid and large-scale, low-cost fabrication of microlens arrays on different substrates [10,15]. However, the preparation of the mold is still a time-critical step that greatly limits the overall process efficiency, especially when small custom systems or rapid prototyping are required. The soft lithography process based on patterned liquid molds provides an idea for inexpensive mold manufacturing [16,17,18]. The rapid and low cost manufacturing of molds for large-sized, small-diameter microlenses remains a challenge. Especially when the diameter of the microlens is required to be less than 50 microns.

EHD printing is one of the most promising micro-nano additive manufacturing technologies with its unique advantages of low cost and high resolution [19,20,21]. It enables the direct, additive patterning of materials with a resolution that can extend below 100 nm, and can print drop on demand. It has been applied to manufacture flexible electronics, sensors, transistors, microfluidic chip, and so on [22,23,24,25,26,27]. Therefore, it is promising to be used to manufacture sub-micron diameter microlens and microlens array molds quickly and cost-effectively. However, if the microlens array is fabricated by directly printing the UV crosslinked material using EHD, the coffee ring effect is often caused by the uneven evaporation rate [28], which affects the optical performance of the microlens.

In this work, we propose a new method for fabricating microlens arrays that combines the advantages of soft lithography and EHD printing. We use a drop-on-demand EHD printing technique to print glycerin liquid on the surface of ITO glass. The glycerol liquid droplets printed on the ITO glass substrate are hemispherical due to surface tension, thus forming microlens liquid mold. The PDMS soft lithography mold is then quickly fabricated using a liquid mold. Finally, the desired micro-optical device can be fabricated on any substrate using a PDMS soft lithography mold. Therefore, our methods allows us to rapidly and cost-effectively fabricate molds with small diameters by exploiting the advantages of EHD printing, while maintaining the parallel nature of soft-lithography and its capacity to generate microlenses of different materials. We also discuss the effects of the main process parameters on the stability of the printing process and the diameter of the droplets.

## 2. Materials and Methods

### 2.1. Chemicals and Materials

The 80 vol% glycerol solution (China Pharmaceutical Group Co., Ltd., Shanghai, China) was selected to be the ink materials for EHD printing the micro-droplet array. Sylgard 184 elastomer base and curing agent for PDMS were purchased from Dow Corning (Midland, MI, USA). The weight ratio of silicone elastomer to curing agent is 10 to 1. The UV-curable prepolymer (X3016, Valigoo Co., Ltd., Dongguan, China) is used as the material for replicating microlens.

### 2.2. EHD Printing System

**Figure 1:**
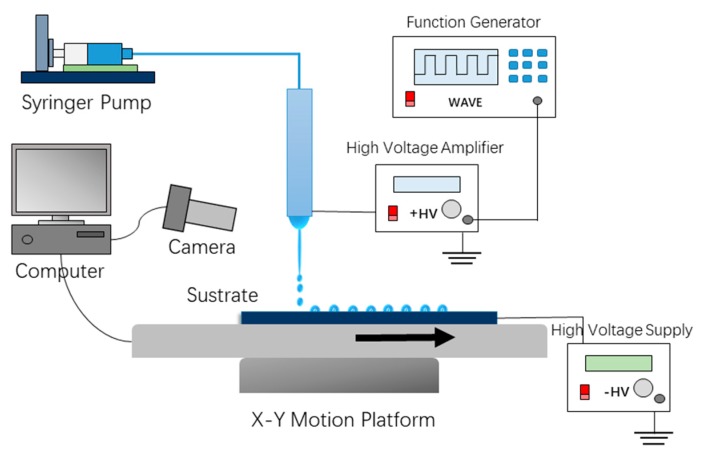
Schematic diagram of electrohydrodynamic (EHD) drop-on-demand printing systems with bias voltage.

The schematic diagram of the experimental EHD printing system for printing liquid microdroplets is shown in Figure 1. A dispensing nozzle (inner diameter 60 μm and external diameter 190 μm) was adopted as an electrode, and the collector was an ITO glass substrate fixed to an XYZ translation system (QZNT-M07, Lepton Inc., Foshan, China) controlled by computer. A precise syringe pump (TJ-2A, Lange, Inc., Baoding, China) feeds the syringe at a controllable flow rate. The distance between dispensing nozzle and collector was set to a range of 800 μm to 1200 μm. In this work, the typical distance was set to 800 μm. The high voltage pulse generator is connected to the nozzle. It consists of a function signal generator (RIGOL-DG4102) and a high voltage amplifier (HVA-103NP6, Tianjin Shenghuo Inc., Tianjin, China). The function signal generator produces a square wave with adjustable frequency and duty cycle, which will then be amplified by the high voltage amplifier. Typical applied voltage, duty cycle, frequency are 2 kV, 20%, and 100 Hz, respectively. A high-voltage power supply (DW-P403-1AC, Tianjin Dongwen Inc., Tianjin, China) with the anode connected to the ITO glass to supply bias voltage. These devices are used to generate electric fields required for EHD printing. Unlike ordinary drop on demand EHD printing, in addition to applying a pulse voltage to nozzle, a negative bias voltage is applied to the ITO glass collector. The typical bias voltage value in this work is −1 kV. The biased voltage helps to generate droplets at low pulse voltages and deposit on the surface of the ITO glass substrate.

### 2.3. PDMS Mold and Microlens Fabrication

**Figure 2:**
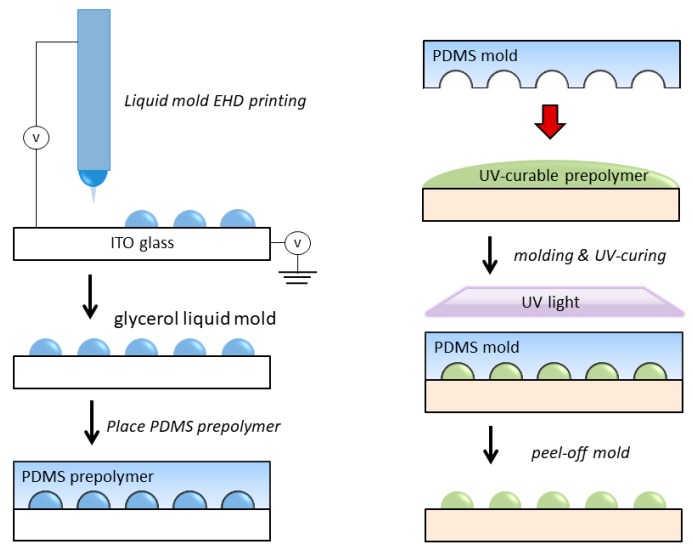
Schematic illustration of the process for fabricating the microlens array replicated films. (**a**) A 2D concave polydimethylsiloxane (PDMS) mode is prepared by combining EHD printing and soft lithography technology and (**b**) a microlens array UV-curable polymer replicated film is prepared by solution casting.

Before the experiment, the ITO glass substrate was ultrasonically cleaned in acetone for 20 min and then dried with air flow. Glycerol solution was first sprayed onto the ITO glass by an EHD printing process. The microdroplets form liquid mold on the surface of ITO glass. The spacing, diameter, and volume of the microdroplets liquid mold can be controlled by processing parameters. The PDMS mold for the microlens fabrication was obtained by casting PDMS mixture (the weight ratio of silicone elastomer to curing agent is 10 to 1) into the ITO glass that contained the EHD printed glycerol micro droplets liquid mold. The cast PDMS was then placed in a vacuum oven at 70 °C for 3 h to fully crosslink it to obtain a PDMS microlens mold. Then take it out, rinse off the surface glycerin, and then dry in an oven for 1 h. The UV crosslinked resin was cast on the surface of any substrate, and then covered with a PDMS mold for solution casting, and irradiated with a 36 W UV lamp (Jmofo, Guangzhou, China) for 3 min. Finally, the PDMS mold was peeled off to obtain a microlens array formed by curing the UV resin (as shown in Figure 2).

### 2.4. Morphological and Optical Characterization

The diameter and shape of the printed microdroplets as well as the PDMS mold and microlens replicas were measured by a measuring microscope (Nikon MM-400, Kanagawa, Japan). The imaging performance of the microlens was tested by a projection experiment, and an image formed by the microlens was observed using an optical microscope (OPTEC-BDS400, Chongqing, China).

## 3. Results and Discussion

Understanding the process of jet formation and determining the key parameters that affect the process are critical to high resolution, uniform, and reproducible printing. In order to obtain a good morphology and meet the requirements of the microlens liquid mold, we explored the drop on demand EHD printing process of printing glycerol solution.

We first explored the parameters that affect the jet formation. The pulse jet formation process is strongly dependent on the distance between nozzle and collector, glycerin solution concentration, pulse voltage, and bias voltage.

The concentration will significantly affect the conductivity and viscosity of the solution, which will have a large impact on the stability of the printing process. When the concentration of the aqueous glycerin solution is high, the viscosity is large and the electrical conductivity is weak, and it is difficult to form a stable pulse jet. When the concentration is too low, the conductivity is enhanced and the viscosity is lowered, which causes a large number of tiny satellite droplets around the main droplet. The printing process is converted into electrospray [29], so that on-demand printing cannot be performed. Therefore, in this work, we chose 80% glycerol solution as the printing material, with the suitable viscosity and no atomization during the printing process.

Electric field is a key factor affecting the formation of a stable pulse jet. It depends on the distance from the needle to the collecting plate and the type and magnitude of the voltage applied to the needle. When the voltage is fixed, if the distance from the needle to the collecting plate is too large, the electric field strength is insufficient to form a jet. However, if the distance is too small (less than 300 μm), corona discharge would occur and prevent the generation of jets. Therefore, setting a suitable distance is critical to the stability of droplet ejection. In this work, the distance was set to a range of 800 μm to 1200 μm to overcome the corona discharge.

**Figure 3:**
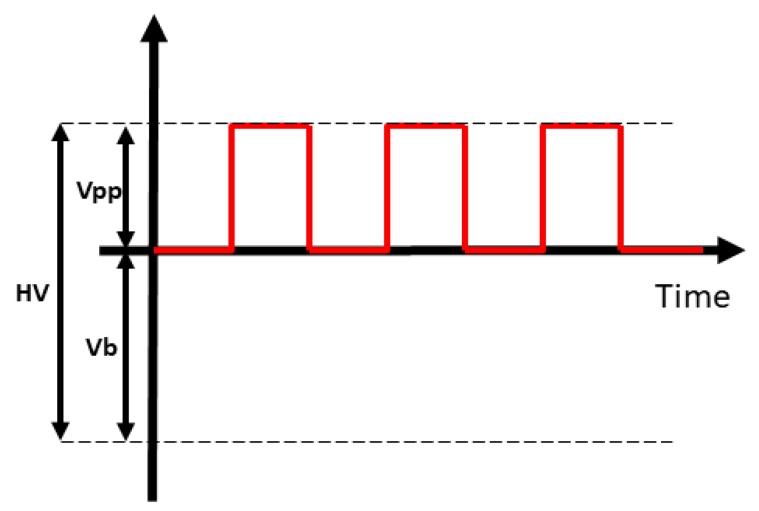
Schematic diagram of voltage waveform. The high voltage (HV) applied to the nozzle is composed of a bias voltage (Vb) and a pulse voltage (Vpp).

When a conventional pulse voltage is applied to the needle for EHD ejection, the lower voltage is insufficient to form a stable pulse jet due to the higher viscosity of the aqueous glycerin solution. However, increasing the pulse voltage or lowering the viscosity of the solution will lead to other undesirable consequences such as corona discharge or a large number of satellite drops. Therefore, in order to achieve a stable on-demand droplet printing of an aqueous glycerin solution, we applied a bias voltage of −1 kV to the ITO glass collecting plate, as shown in Figure 1 and Figure 3. This is different from most of the previous works. With the help of the bias voltage, the droplets are stretched to form a Taylor cone and reach a specific angle required to form the jet. The drop on demand injection is then achieved under the action of the pulsed electric field. The bias voltage therefore plays a key role in achieving the on-demand droplet ejection of the higher viscosity aqueous glycerol solution. Through several experiments, we found that a bias voltage range of 1 kV to 1.5 kV with a height range of 800 μm to 1200 μm can help generate drop on demand jets and uniform microdroplets.

We also explored the parameters that affect the diameter of the microdroplets dot pattern. It is mainly affected by pulse voltage frequency, pulse duty cycle, flow velocity of solution, and pulse voltage amplitude.

The frequency of the droplet ejection is equal to the frequency of the pulse voltage. The spacing of the two droplets ejected onto the substrate can be precisely adjusted by controlling the pulse frequency and the moving speed of the collecting plate. The spacing of the two dot *D_p_* can be calculated by

(1) $$D_{p}=\frac{V_{plate}}{f}$$

where *V_plate_* is the collecting plate velocity, *f* is the frequency of the pulse voltage.

Stinger and Derby [30] studied the formation of deposit by inkjet printing of a series of ink. Based on Stinger’s work, Jaehong Park et al. [31] and Lei Xu et al. [32] proposed the diameter prediction models for EHD printing. According to their models, droplets deposited on a substrate can be calculated as

(2) $$D_{dot}=D_{drop}{\left\{\frac{4\sin\theta^{3}}{{\left(1-\cos\theta \right)}^{2}(2+\cos\theta )}\right\}}^{\frac{1}{3}}$$

where *D_dot_* is the spherical cap (dot pattern) diameter, *D_drop_* is the droplet diameter, and *θ* is the contact angle.

The drop diameter can be calculated as follows

(3) $$D_{drop}={\left(\frac{6}{\pi }V_{drop}\right)}^{\frac{1}{3}}$$

(4) $$V_{drop}=Q\times T$$

(5) $$T=\frac{d}{f}$$

where *V_drop_* is the total droplet volume ejected from the Taylor cone during each pulse period. *Q* is the flow rate, *T* is pulse width time, *d* is the duty ratio, and *f* is the pulse frequency.

Therefore, deposited pattern diameter can be expressed as [27]

(6) $$D_{dot}={\left\{\frac{24\sin\theta^{2}Qd}{\pi f{\left(1-\cos\theta \right)}^{2}(2+\cos\theta )}\right\}}^{\frac{1}{3}}$$

**Figure 4:**
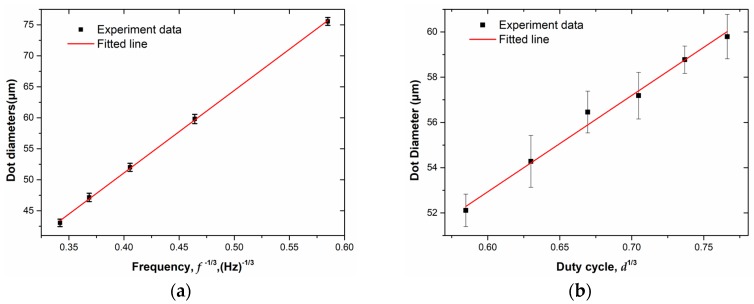
(**a**) The dot diameters as a function of dimensionless pulse voltage frequency *f*^−1/3^. The applied voltage, bias voltage, duty cycle, flow rate, and the distance between spinneret and collector are 2 kV, −1 kV, 20%, 0.15 μL/min, and 0.8 mm, respectively. (**b**) The dot diameters as a function of dimensionless duty cycle *d*^1/3^. The applied voltage, bias voltage, frequency, flow rate, and the distance between spinneret and collector are 2 kV, −1 kV, 10 Hz, 0.15 μL/min, and 0.8 mm, respectively.

As the pulse frequency increases, the time during which each injection cycle voltage acts on the Taylor cone becomes shorter, the accumulated charge and the volume of the solution decrease, and thus the diameter of the droplet decreases. Therefore the frequency of the pulse voltage will significantly affect the dot pattern diameter, as shown in Figure 4a. The scaling law can be written as

(7) $$D_{dot}∼f^{-\frac{1}{3}}$$

We kept the pulse period fixed and increased the voltage duty cycle and found that the droplet diameter increased. When the duty time increased, the volume of the solution and the accumulated charges on the taylor cone increased, so that the repulsive force became stronger, which resulted in a larger diameter microdroplets. Therefore, the duty cycle of the pulse voltage per injection is a key factor affecting the droplet diameter and dot pattern diameter, as shown in Figure 4b. The dot diameters as a function of dimensionless duty cycle *d*^1/3^.

(8) $$D_{dot}\sim d^{\frac{1}{3}}$$

**Figure 5:**
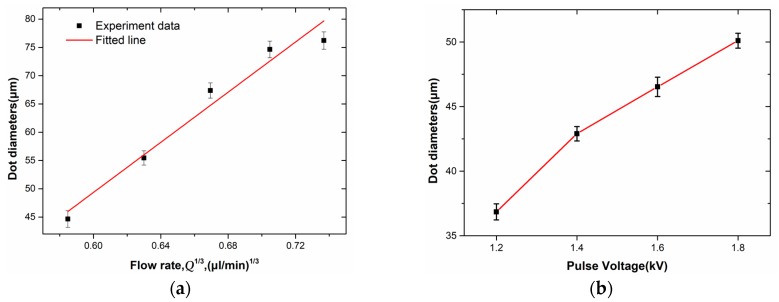
(**a**) The relationship between dot diameters and dimensionless flow rate *Q*^1/3^. The applied voltage, bias voltage, duty cycle, frequency, and the distance between spinneret and collector are 2 kV, −1 kV, 20%, 100 Hz, and 1 mm, respectively. (**b**) The relationship between dot diameters and pulse voltage. The bias voltage, duty cycle, frequency, flow rate, and the distance between spinneret and collector are −1.5 kV, 20%, 20 Hz, 0.15 μL/min, and 1 mm, respectively.

Figure 5a shows the effect of flow rate on droplet diameter, which reveals that the diameter of the droplet increases with increasing flow rate. Because the flow rate will affect the angle of the Taylor cone and the volume of solution accumulated during each pulse duty cycle, thereby affecting the diameter of the droplet. The scaling law between *D_dot_, Q* is

(9) $$D_{dot}∼Q^{\frac{1}{3}}$$

We further studied the effects of pulse voltage on droplet diameter. Figure 5b shows the effect of the applied pulse voltage amplitude on the droplet diameter at a flow rate of 0.2 μL/min, which reveals that the diameter of the droplet increases as the pulse voltage increases. The increase in the amplitude of the pulse voltage increases the electric field force and Coulomb repulsive force for each injection cycle, increasing the stretch force acting on the Taylor cone, thereby increasing the diameter of the ejected droplet.

**Figure 6:**
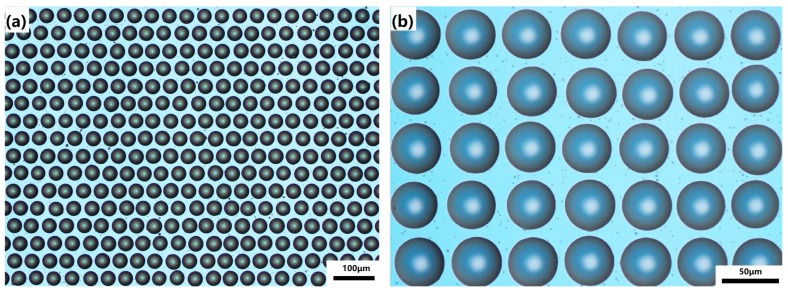
(**a**,**b**) Optical microscope image of the microlens liquid mold produced by EHD printing.

**Figure 7:**
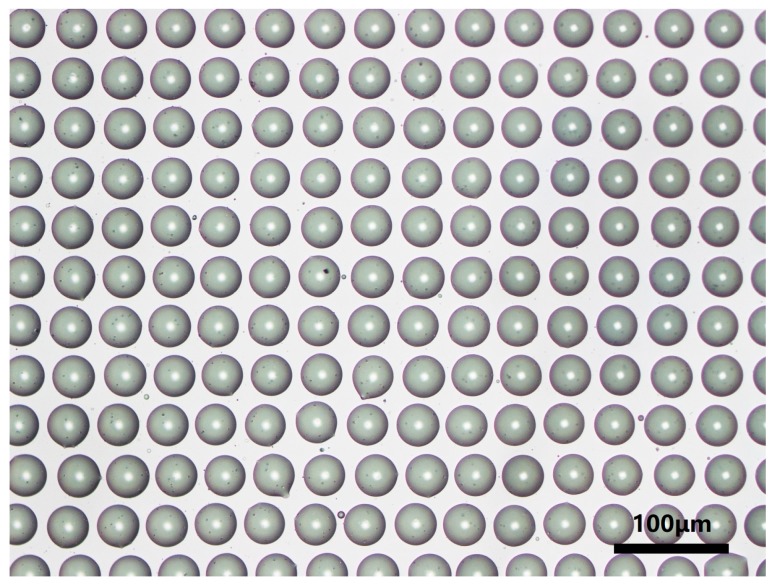
Optical microscope image of the final fabricated microlens.

Finally, we combined the PDMS soft lithography process to prepare microlenses based on liquid molds. The PDMS mixture (the weight ratio of silicone elastomer to curing agent is 10 to 1) was slowly poured onto the liquid microlens mold (as shown in Figure 6). Since glycerin has a certain viscosity and can adhere well to the surface of the ITO glass, it can still maintain a good morphology when the PDMS is slowly poured. However, this process must be carefully done, paying attention to the rate of pouring, which would otherwise cause the liquid mold to be washed away. The cast PDMS was then heat cured in a vacuum oven at 70 °C for 3 h to obtain a PDMS microlens mold. Then it is taken out and the surface glycerin is rinsed off and then dried in an oven for 1 h. The UV crosslinked resin was cast on the surface of PET substrate, and then covered with a PDMS mold for solution casting, and irradiated with a 36 W UV lamp for 3 min. Finally, a microlens array of UV curable resin was obtained using solution casting PDMS mold, as shown in Figure 7.

**Figure 8:**
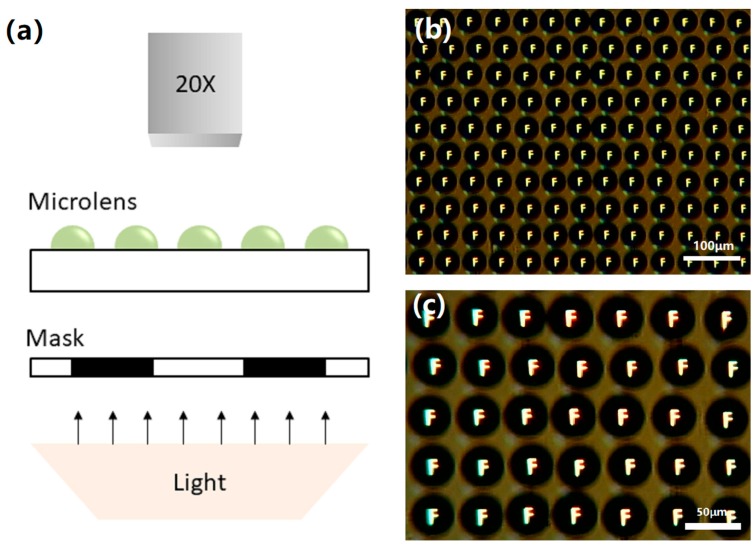
The imaging performance of the microlens. (**a**) Schematic illustration of the testing setup for the microlens. (**b**,**c**) Image of an array of inverted “F”s imaged through microlens array. The inverted images are clear which shows good uniformity among the neighboring microlenses.

We have characterized the obtained micro lenses array. Optical micrographs of representative lenses clearly illustrate the quality of the fabricated structure. To demonstrate the imaging performance of micro lenses array, an optical digital microscope (OPTEC-BDS400) was used for micro lenses array projection experiments. The mask we inserted with the letter “F” is printed on a black paper between the bright light source and the micro lenses, as shown in the Figure 8a. Finally, the reduced letters are projected onto a digital microscope. Figure 8b,c shows a clear image of the letter “F” on the micro lenses array. These indicate that micro lenses array has good imaging performance.

## 4. Conclusions

In summary, we propose a simple, economical method to rapidly fabricate microlens arrays based on liquid mold soft lithography processes. Compared to other inkjet printing technologies, our DOD EHD process can print smaller droplets at a higher speed. In addition, once the PDMS concave microlens mold is fabricated, it can be reused for microlens replication without having to go through the entire process again. We also studied the process parameters that affect the stability of the printing process and the diameter of the printed micro droplets. By applying a biased DC voltage on the collector plate, it helps to form a stable pulse jet, avoiding satellite drops or corona discharge. This work was expected to promote the application of EHD printing technologies in the fields of manufacturing of micro system.
